# How ontology can be used to achieve semantic interoperability in healthcare

**DOI:** 10.1093/eurpub/ckac129.363

**Published:** 2022-10-25

**Authors:** S Das, P Hussey

**Affiliations:** Centre for eIntegrated Care, Dublin City University, Dublin, Ireland

## Abstract

Semantic interoperability allows machines to share, interpret and use data without ambiguity. Semantic Interoperability is a major concern in healthcare (c.f. the EU commission 2021 report on electronic record exchange formats). The lack of interoperability with regard to electronic health record (EHR) leads to fragmentation and a lower quality of cross-institution and cross-border healthcare. The simple choice of an interchange language (HL7, FIHR etc.)is not sufficient to ensure interoperability. Healthcare interoperability is associated with multi-level and multi-sectoral complexity, and this cannot be addressed without consideration of a range of people and needs, from application design to knowledge sharing. Each transaction needs to be defined in unambiguous details as part of a complete, consistent, coherent, and machine-readable set of specifications for interoperability between the machines to minimize any potential error. We propose a systematic process to achieve healthcare interoperability, working with healthcare professionals starting from design level to implementation level. In our seminar we will explain with examples how ontology can be used to achieve semantic interoperability in healthcare. Technical requirements, including the choice of tools (e.g. Protégé); data base (e.g. GraphDB); data model (i.e. Web Ontology Language 2 (OWL2); formal specification (i.e. Description logics (DL)); and the right syntax (RDF/XML). Will be introduced.

